# Genotype-Specific Interaction of Latent TGFβ Binding Protein 4 with TGFβ

**DOI:** 10.1371/journal.pone.0150358

**Published:** 2016-02-26

**Authors:** Kay-Marie Lamar, Tamari Miller, Lisa Dellefave-Castillo, Elizabeth M. McNally

**Affiliations:** 1 Department of Human Genetics, The University of Chicago, Chicago, Illinois, United States of America; 2 Center for Genetic Medicine, Northwestern University Feinberg School of Medicine, Chicago, Illinois, United States of America; University of Miami School of Medicine, UNITED STATES

## Abstract

Latent TGFβ binding proteins are extracellular matrix proteins that bind latent TGFβ to form the large latent complex. Nonsynonymous polymorphisms in *LTBP4*, a member of the latent TGFβ binding protein gene family, have been linked to several human diseases, underscoring the importance of TGFβ regulation for a range of phenotypes. Because of strong linkage disequilibrium across the *LTBP4* gene, humans have two main *LTBP4* alleles that differ at four amino acid positions, referred to as IAAM and VTTT for the encoded residues. VTTT is considered the “risk” allele and associates with increased intracellular TGFβ signaling and more deleterious phenotypes in muscular dystrophy and other diseases. We now evaluated *LTBP4* nsSNPs in dilated cardiomyopathy, a distinct disorder associated with TGFβ signaling. We stratified based on self-identified ethnicity and found that the *LTBP4* VTTT allele is associated with increased risk of dilated cardiomyopathy in European Americans extending the diseases that associate with *LTBP4* genotype. However, the association of *LTBP4* SNPs with dilated cardiomyopathy was not observed in African Americans. To elucidate the mechanism by which *LTBP4* genotype exerts this differential effect, TGFβ’s association with LTBP4 protein was examined. LTBP4 protein with the IAAM residues bound more latent TGFβ compared to the LTBP4 VTTT protein. Together these data provide support that *LTBP4* genotype exerts its effect through differential avidity for TGFβ accounting for the differences in TGFβ signaling attributed to these two alleles.

## Introduction

Latent TGFβ binding protein 4 (LTBP4) is part of a family of extracellular proteins including LTBPs 1–3 as well as the fibrillins [1, 2]. Members of this family are characterized by the presence of multiple epidermal growth factor-like repeats, and conserved 8-cysteine domains. *LTBP4* is expressed at high levels in the heart, skeletal and smooth muscle but also shows lower level expression in other tissues [1, 2]. Latent TGFβ is held in an inactive state in the extracellular matrix as part of a large latent complex (LLC) consisting of TGFβ, its latency associated peptide and LTBP. The regulation of TGFβ is tightly controlled, and in order to become active, TGFβ must be free of both latency associated peptide and LTBP. Proteolysis of LTBP or force-induced release of TGFβ by LTBP results in liberation of the active TGFβ dimer, engagement of cell surface receptors and induction of intracellular downstream signaling [3, 4]. In addition to regulating the release of TGFβ, LTBP also participates in the assembly and secretion of TGFβ [5, 6].

TGFβ is a multifunctional molecule that regulates growth, development, and response to injury. Three TGFβ isoforms, TGFβ1, 2 and 3, are highly conserved, with between 70–80% identity in their active domain. Despite high similarity, the TGFβ isoforms have different spatiotemporal expression during development, as well as during wound healing [7, 8]. In wound healing, these TGFβ family members have been implicated in inflammation, proliferation, and tissue remodeling [9]. TGFβ family members also directly regulate matrix deposition and fibrosis by stimulating production of components such as fibronectin and collagen and simultaneously downregulating matrix-degrading proteases [10–12]. Excessive fibrosis and TGFβ signaling are found in a number of chronic pathological processes including muscular dystrophy, liver cirrhosis, and idiopathic pulmonary fibrosis [13, 14]. In these disorders, increased or “hyper-TGFβ” signaling leads to accumulated matrix-associated proteins, scarring and fibrosis. TGFβ also undergoes auto-induction, which further amplifies its effects [8].

Non-synonymous single nucleotide polymorphisms (SNPs) in *LTBP4* have been associated with pathogenicity in several distinct human disorders. In humans with Duchenne Muscular Dystrophy (DMD), *LTBP4* genotype has been associated with prolonged ambulation in multiple cohorts [15–17]. In chronic obstructive pulmonary disease, *LTBP4* SNPs have been linked to improved exercise capacity, including increased six-minute walk test distance and greater maximum work capacity [18]. SNPs in *LTBP4* have also been associated with reduced expansion of abdominal aortic aneurysm, and less aggressive tumors in colorectal cancer [18–20].

Dilated cardiomyopathy (DCM) is genetically heterogeneous and is often characterized by fibrosis and abnormal TGFβ signaling [21, 22]. Polymorphisms in *TGFB1* have been associated with heart failure caused by DCM, and TGFβ is upregulated in the plasma and myocardium of DCM patients [23–26]. In order to assess whether *LTBP4* contributes to DCM disease risk, we now genotyped *LTBP4* polymorphisms in cases and controls and found an overabundance of risk alleles in European American DCM subjects. To assess the biological effects of the two most common *LTBP4* alleles in the human genome, we co-expressed LTBP4 protein along with TGFβ. We found that LTBP4 protein expressed with the protective four amino acids, IAAM, associated with more TGFβ compared to LTBP4 expressing the deleterious residues VTTT. In this model, decreased affinity of *LTBP4* for latent TGFβ accounts for the increased TGFβ and TGFβ signaling seen with the VTTT allele. Together these findings provide a molecular mechanism by which *LTBP4* modifies chronic fibrotic disorders.

## Results

### *LTBP4* SNPs are in linkage disequilibrium and associate with disease phenotypes linked to TGFβ signaling

**Fig 1:**
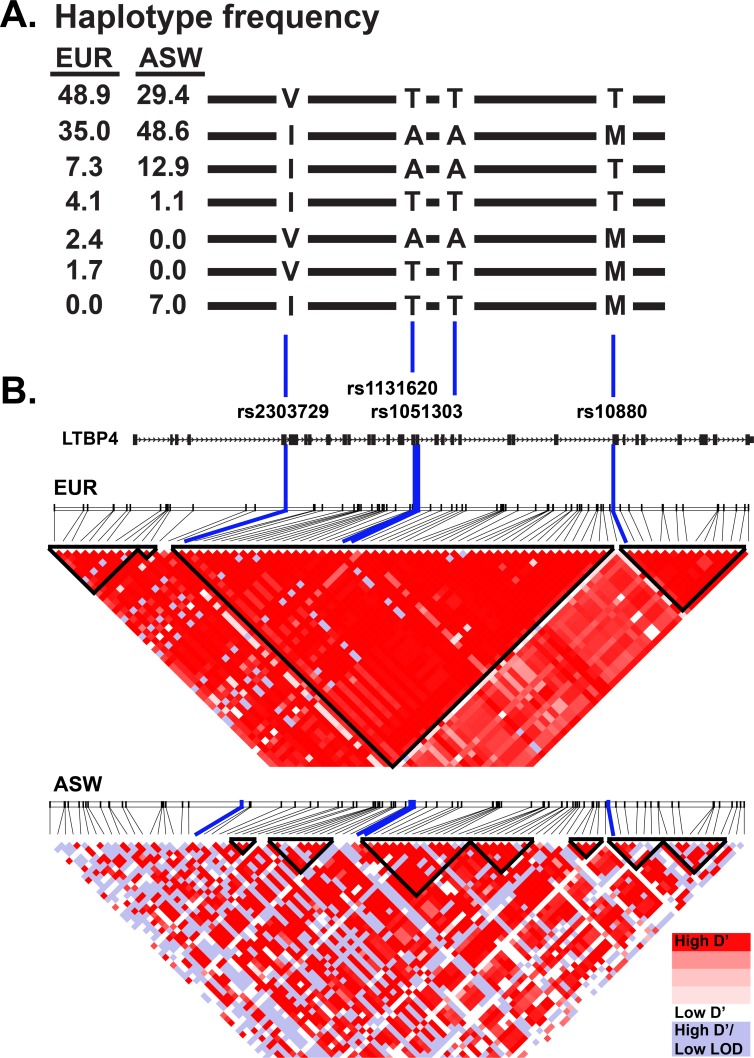
Linkage disequilibrium across the *LTBP4* locus varies with ethnicity. **A.** Haplotype frequency of *LTBP4* single nucleotide polymorphisms rs2303729, rs1131620, rs1051303, and rs10880. Frequencies are shown for 1000 Genomes EUR (European American) and 1000 Genomes ASW (African Americans from Southwestern United States). The two major *LTBP4* haplotypes are referred to as VTTT and IAAM for the specific amino acid changes, and together these represent 83.9 and 78 percent of the alleles in European Americans (EUR) and African Americans (ASW), respectively. **B.** Linkage disequilibrium map of *LTBP4* locus in European Americans (1000 Genomes EUR) and African Americans (1000 Genomes ASW). Linkage disequilibrium blocks (black triangles) are designated. The map shows the high level of linkage disequilibrium in European Americans and the comparatively disrupted linkage disequilibrium in African Americans.

There are several common nonsynonymous SNPs in the human *LTBP4* gene, and these include rs2303729, rs1131620, rs1051303, and rs10880 [27, 28]. These SNPs are in strong linkage disequilibrium forming two major haplotypes (Fig 1). One haplotype encodes the amino acids Valine, Threonine, Threonine, and Threonine, referred to as VTTT, and the other encodes Isoleucine, Alanine, Alanine, and Methionine, referred to as IAAM. The frequency of the VTTT haplotype in Europeans is 49%, and the IAAM allele frequency is 35%, while minor haplotypes, deviating at one or more of the SNPs contribute to the remaining 16% (Fig 1).

**Fig 2:**
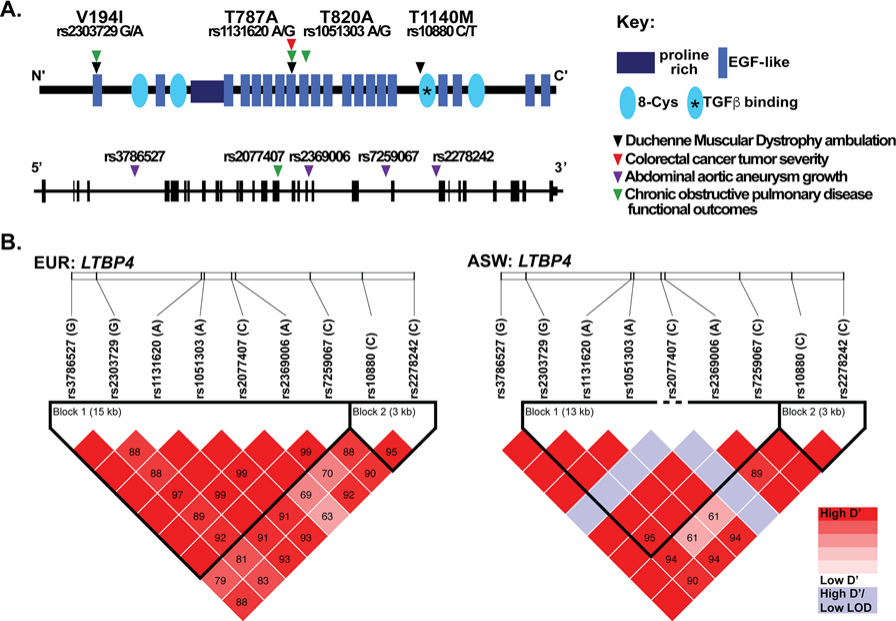
Single nucleotide polymorphisms (SNPs) in *LTBP4* modify many human diseases. **A.** The protein schematic of human LTPB4 protein, with arrowheads indicating the position of the non-synonymous SNPs (nsSNPs) that have been reported to associated with disease severity [15–20]. The *LTBP4* genomic locus is shown below with the vertical black bars representing exonic regions and horizontal black line representing intronic portions. Arrowheads indicate intronic and synonymous SNPs in *LTBP4* that modify human disease. **B.** Linkage disequilibrium map from Haploview of nine *LTBP4* SNPs previously associated with disease phenotypes in chronic obstructive pulmonary disease, colorectal cancer, abdominal aortic aneurysm, and Duchenne muscular dystrophy. This map was created using data from the 1000 Genomes EUR (European American) population or the ASW population (African Americans from Southwestern United States). Pair-wise D’ values are shown in the boxes. Colors are based on the standard D’/LOD option in the Haploview software.

The IAAM allele was associated with prolonged ambulation in patients with DMD, a progressive muscle wasting disorder characterized by extensive muscle fibrosis [16]. Of the four SNPs that constitute the *LTBP4* haplotype, rs10880 was the most significantly associated with prolonged ambulation [16]. In addition to DMD, *LTBP4* SNPs have also been associated with other disease phenotypes (Fig 2A). The nsSNP 1131620 (Thr787Ala) was associated with more aggressive tumors in colorectal cancer [20]. An enhanced growth rate of abdominal aortic aneurysms was linked to several intronic SNPs in *LTBP4* including rs3786527, rs2369006, rs7259067, rs2278242, and a borderline association with the presence of abdominal aortic aneurysms was found for synonymous SNP rs2077407 [19]. These intronic and synonymous SNPs are in high linkage disequilibrium with the VTTT haplotype (Fig 2B). In a separate study, reduced work capacity, lower exercise tolerance, and shorter 6-minute walk test distance in chronic obstructive pulmonary disease were associated with SNPs rs2303729, rs1131620, rs1051303, and rs2077407 [18]. In each of these cases, the *LTBP4* haplotype that specifies the VTTT residues was considered the risk allele for the phenotype of interest, and correspondingly the IAAM haplotype was considered the protective allele. Fibroblasts with the VTTT allele were found to have enhanced TGFβ signaling, while the IAAM allele was associated with reduced TGFβ signaling [16]. Together these data support that increased TGFβ signaling associates with increased disease burden in these disorders.

### The *LTBP4* VTTT allele is associated with DCM in European Americans

Aberrant TGFβ signaling is also a feature of DCM, a disorder frequently associated with fibrosis [21, 22]. In order to identify genes that are co-expressed with *LTBP4*, *LTBP4* was evaluated using the Co-Regulation Database (CORD) [29]. *LTBP4* co-regulated genes were highly expressed in the heart (p = 2.4x10^-12^) and over representative for the DCM KEGG pathway (p = 0.0007), positioning *LTBP4* in the etiology of DCM (S1–S3 Tables). Furthermore, mice lacking the short isoform of *Ltbp4* (*Ltbp4S*^-/-^) develop cardiomyopathy with biventricular dilatation [30]. In order to assess the effect of *LTBP4* genotype on DCM risk, we genotyped *LTBP4* SNPs in DCM subjects and unaffected controls. We genotyped *LTBP4* SNPs in African American (AA) and European American (EUR) DCM patients and compared each group to ethnicity-matched controls. Disease severity was comparable between the two cohorts of DCM patients. The left ventricular ejection fraction (LVEF) was 27.07±10.03 in AA DCM and 28.01±6.94 in EUR DCM (p = 0.49), and the age at which these measurements were made was comparable 42.06±1.50 years versus 44.87±1.60 years (p = 0.228).

The frequency of the VTTT allele was increased in DCM patients compared to controls in EUR DCM (Table 1). The G allele of rs2303729 was found at a frequency of 0.712 in EUR DCM patients compared to 0.514 in healthy controls (OR = 2.259, p<0.0001). Similarly, the A allele of rs1131620 and the A allele of rs1051303, were found at frequencies of 0.720 in EUR DCM patients and 0.564 or 0.553 in controls, respectively (OR = 2.004, p = 0.0004; OR = 2.090, p = 0.0002). Finally, the C allele of rs10880 was found at a frequency of 0.721 in EUR DCM patients and 0.609 in ethnicity-matched controls (OR = 1.653, p = 0.0176). In AA with DCM, no association was observed between *LTBP4* nsSNPs and DCM status (range of p values from 0.200 to 0.996, depending on SNP, Table 2).

**Table 1:** *LTBP4* nsSNPs are associated with DCM in European Americans.

		SNP Reporting frequency of “Risk” Allele (VTTT)			
		rs2303729 (G)	rs1131620 (A)	rs1051303 (A)	rs10880 (C)
Population	n	V194I	T787A	T820A	T1140M
DCM Patients (EUR)	122	.712	.72	.72	.721
1000 Genomes EUR	1006	.536	.548	.548	.608
Coriell Controls	200	.515	.551	.551	.613
NHLBI Exome Sequencing Project (ESP)	8600	.546	.578	.582	-
Exome Aggregation Consortium (ExAC) (EUR)	73354	.514	.564	.553	-
Controls combined^†^		.514	.564	.553	.609
Odds Ratio (95% CI)		2.259 (1.530–3.334)	2.004 (1.349–2.978)	2.090 (1.407–3.107)	1.653 (1.087–2.511)
χ^2^ p-value		<0.0001	0.0004	0.0002	0.0176

DCM = Dilated cardiomyopathy, EUR = 1000 Genomes European super population, CI = confidence interval.

† ExAC and Coriell controls were combined for rs2303729, rs1131620, and rs1051303. 1000 Genomes EUR and Coriell controls were combined for rs10880. Rs10880 has low sequencing coverage in exome sequencing in ESP and ExAC.

**Table 2:** *LTBP4* SNPs have similar frequency in African American DCM subjects and controls.

		SNP Reporting frequency of “Risk” Allele (VTTT)			
		rs2303729 (G)	rs1131620 (A)	rs1051303 (A)	rs10880 (C)
Population	n	V194I	T787A	T820A	T1140M
DCM Patients (African American)	280	.304	.432	.432	.496
1000 Genomes ASW	122	.303	.385	.385	.434
Coriell Controls	200	.38	.439	.439	.54
Cord Blood Controls	142	-	.433	.431	.537
TRIDOM Controls	396	-	.435	.438	-
NHLBI Exome Sequencing Project (ESP)	4406	.340	.437	.432	-
Exome Aggregation Consortium (ExAC) AFR^§^	10406	.325	.426	.417	-
Controls combined^†^		.341	.435	.432	.511
Odds Ratio (95% CI)		0.8424 (0.6478–1.096)	0.9872 (0.7743–1.259)	1.001 (0.7847–1.276)	0.9715 (0.7184–1.314)
χ^2^ p-value		0.2002	0.9172	0.9960	0.8509

DCM = Dilated cardiomyopathy, ASW = African Americans from Southwestern United States

^§^ExAC AFR-Includes Africans and African Americans and was not included in the p-value calculation.

^†^Coriell controls, Cord Blood controls, TRIDOM controls, 1000 Genomes ASW, and ESP controls were combined for rs2303729, rs1131620, and rs1051303. 1000 Genomes ASW, Coriell controls, Cord Blood Controls, and TRIDOM controls were combined for rs10880. Rs10880 has low sequencing coverage in exome sequencing in ESP and ExAC.

The frequency of *LTBP4* SNPs varies with ethnicity and indeed was observed to vary slightly even among African American control cohorts. Potential admixture differences between subjects and controls led us to genotype two additional control DNA sets from AA populations as well as examine public databases. Adult controls, composed of adults 20–60 years of age from the Chicago area, were genotyped for rs1051303 and rs1131620. Pediatric controls from the same geographic area were genotyped for rs1051303 and rs1131620, and rs10880. No significant differences in allele frequency between African American DCM patients and controls were observed (Table 2). The frequencies in these local populations were compared with public databases of *LTBP4* SNP frequencies including the NHLBI Exome Sequencing Project, which includes 4300 EUR and 2300 AA individuals. The first three *LTBP4* nsSNPS were included in this analysis since their depth of sequencing was adequate to reliably interpret genotype. SNP rs10880 was excluded in this analysis since this region is not adequately covered by exome sequencing. Taking these data into account yields similar *LTBP4* SNP frequencies as seen in the local control cohorts and confirms the association of *LTBP4* SNPs with DCM was only observed for EUR DCM subjects.

### LTBP4 IAAM interacts with TGFβ1 more than LTBP4 VTTT

**Fig 3:**
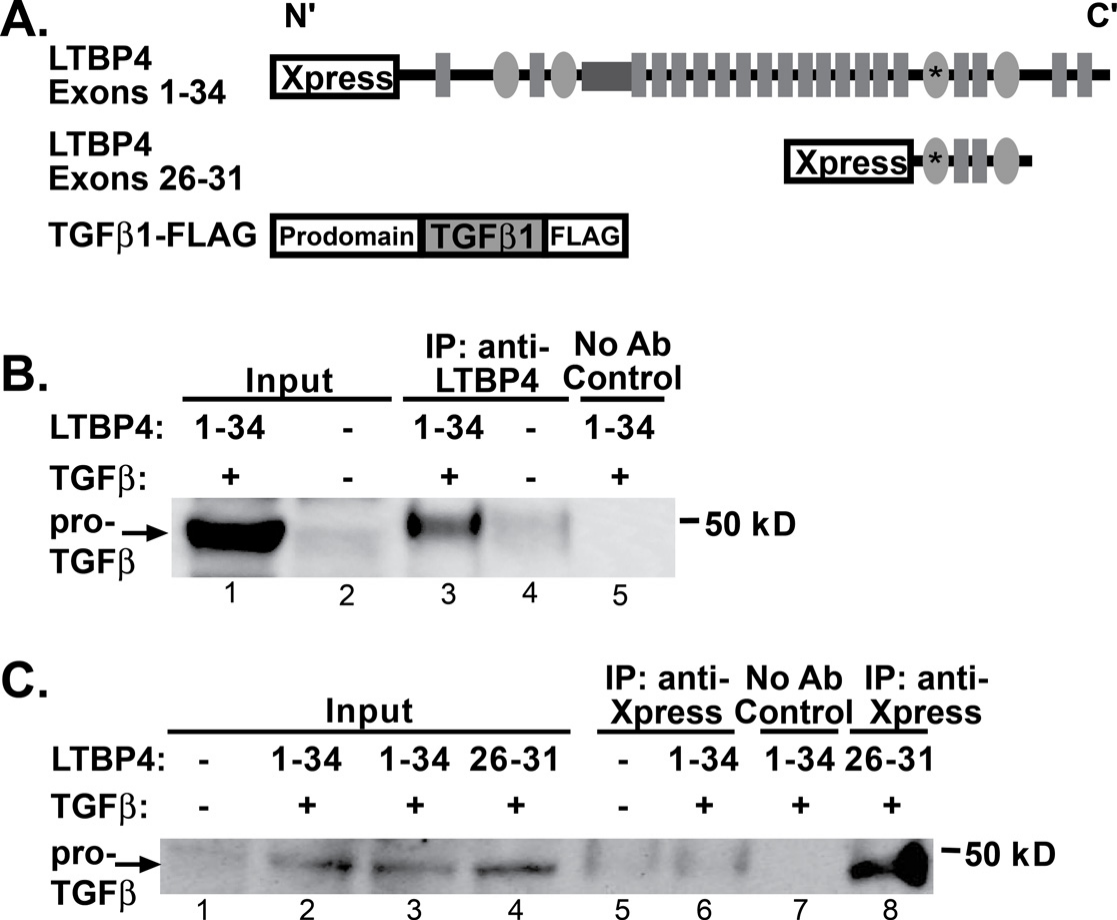
TGFβ1 binds the carboxy terminus of LTBP4. **A.** HEK293T cells were transfected with human constructs to express LTBP4 and TGFβ1. **B.** Immunoprecipitation was performed from cell lysates by immunoprecipitating with an anti-LTBP4 antibody followed by immunoblotting with anti-FLAG antibody, which detected an interaction between LTBP4 and pro-TGFβ1. The immunoblot shows that full length LTBP4 binds TGFβ1. **C.** Co-immunoprecipitation was performed on cell lysates by immunoprecipitating with anti-Xpress antibodies to the epitope tag on LTBP4 followed by immunoblotting with anti-FLAG antibody detecting TGFβ1. The carboxy terminal fragment of LTBP4 associated with TGFβ1. Controls were performed without adding immunoprecipitating antibody.

Latent TGFβ binding proteins regulate the availability of the small latent complex of TGFβ, and increased TGFβ is associated with fibrosis in multiple diseases, including muscular dystrophy [13, 31]. To assess LTBP4’s interaction with latent TGFβ, co-immunoprecipitation experiments were carried out. Human embryonic kidney (HEK) 293T cells were co-transfected with constructs expressing the long isoform of LTBP4 and latent TGFβ1 (Fig 3A). Cells were lysed, and proteins were separated and then immunoprecipitated using an antibody to LTBP4 demonstrating that TGFβ1 associates with LTBP4 (Fig 3B, lane 3). A control experiment without the LTBP4 antibody showed that the interaction with TGFβ was dependent on LTBP4. In order to refine the region of LTBP4 responsible for binding TGFβ1, amino- and carboxy-terminal fragments of LTBP4 were individually expressed and tested. The carboxy-terminal fragment encoded by exons 26–31 of *LTBP4* includes the 3^rd^ and 4^th^ 8-cysteine domains and two epidermal growth factor-like repeats (Ex26-31) (Fig 3A). This fragment of LTBP4 showed robust interaction with TGFβ (Fig 3C, lane 8). The faint 50 kDa band present in the untransfected lane (lane 5) corresponds to low level cross reactivity to the antibody 50 kDa heavy chain used for immunoprecipitation. Cells transfected with full length LTBP4 and TGFβ1 show only the heavy chain contamination (lane 6), but this negative result is attributed to the observation that full length LTBP4 is not efficiently pulled down with the anti-Xpress antibody. The amino-terminal region of LTBP4 did not produce a detectable association with TGFβ1 (data not shown).

**Fig 4:**
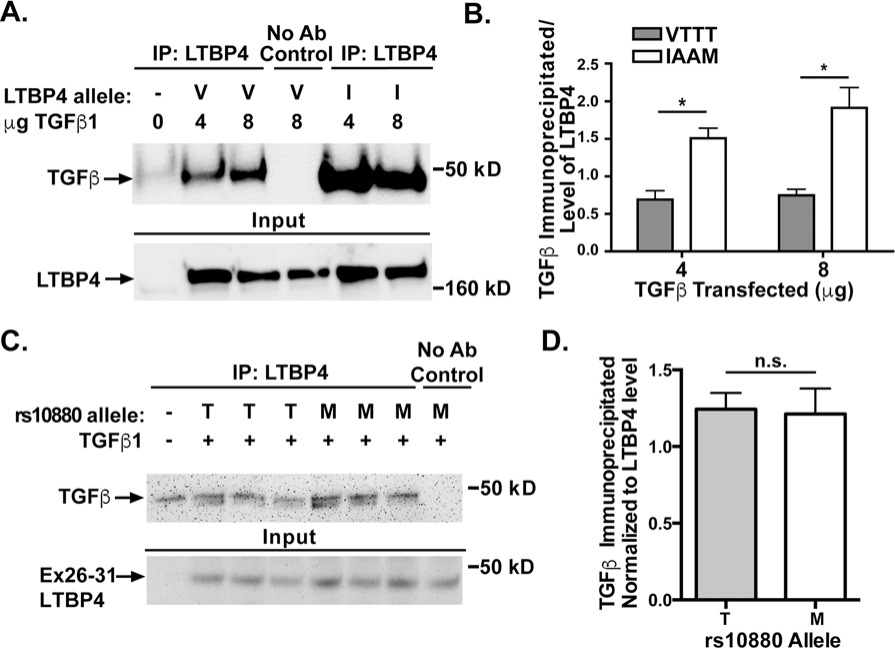
LTBP4-IAAM binds more TGFβ1 than LTBP4-VTTT. **A.** HEK293T cells were co-transfected with constructs expressing pro-TGFβ1 and either LTBP4-VTTT (V) or LTBP4-IAAM (I). Cell lysates were immunoprecipitated using an antibody to LTBP4 and immunoblotted using an antibody to the FLAG tag on TGFβ1. Both LTBP4-IAAM and LTBP4-VTTT bound pro-TGFβ1. **B.** The amount of TGFβ1 immunoprecipitated was quantified and normalized to the amount of LTBP4 expression (experiments performed in triplicate). Cells lines carrying the IAAM protective allele of LTBP4 immunoprecipitate more TGFβ1 than cell lines carrying the VTTT allele, (p = 0.0015). **C.** The carboxy terminal fragment of LTBP4 that contains rs10880 encoding either the “M” or “T” residue was expressed (Ex26-31) and tested for its interaction with TGFβ1. **D.** No significant difference was detected between the reference threonine (T)-containing and the protective methionine (M)-containing fragment of LTBP4 in its ability to interact with TGFβ1.

Fibroblasts with the IAAM allele of *LTBP4* have lower levels of phosphorylated SMAD than cell lines carrying the VTTT allele [16]. In order to understand these differences in TGFβ signaling, LTBP4 proteins expressing these two haplotypes, IAAM and VTTT, were tested for binding to latent TGFβ. HEK293T cells were co-transfected with two different concentrations of plasmid expressing latent TGFβ1 and a construct expressing either the VTTT or IAAM allele of LTBP4 (V and I, respectively, Fig 4). Transfections and immunoprecipitations were done in triplicate. Cell lysates were immunoprecipitated using an antibody to LTBP4 and immunoblotted using an antibody to the FLAG tag on TGFβ1 (Fig 4A). The amount of TGFβ1 immunoprecipitated was quantified and normalized to the amount of LTBP4 expression (Fig 4B). Cells lines expressing the IAAM protective allele of LTBP4 immunoprecipitated more TGFβ1 than LTBP4 with the VTTT allele when transfected with either 4μg TGFβ1 (p = 0.0106) or 8μg TGFβ1 (p = 0.0146). These data are consistent with a model in which the VTTT allele associates less tightly to latent TGFβ, leading to increased available latent TGFβ and therefore increased TGFβ signaling. The rs10880 SNP falls near the TGFβ binding domain of LTBP4, therefore we tested whether this SNP alone would result in differential binding of TGFβ. The exon 26–31 portion of LTBP4 was mutated to include the reference allele of rs10880 (encoding Threonine or T) or the protective allele (encoding Methionine or M). These constructs were transfected into HEK293T cells along with TGFβ1. Cell lysates were immunoprecipitated using an antibody to the Xpress tag on LTBP4, followed by immunoblotting for the FLAG tag on TGFβ1 (Fig 4C). When normalized to LTBP4 protein levels, no significant difference was seen (Fig 4D). This finding does not exclude the possibility that this variant mediates an important role in the interaction, as any single amino acid substitution may need to act in concert with the remaining protein to see this effect.

### LTBP4 binds TGFβ1, TGFβ2, and TGFβ3

**Fig 5:**
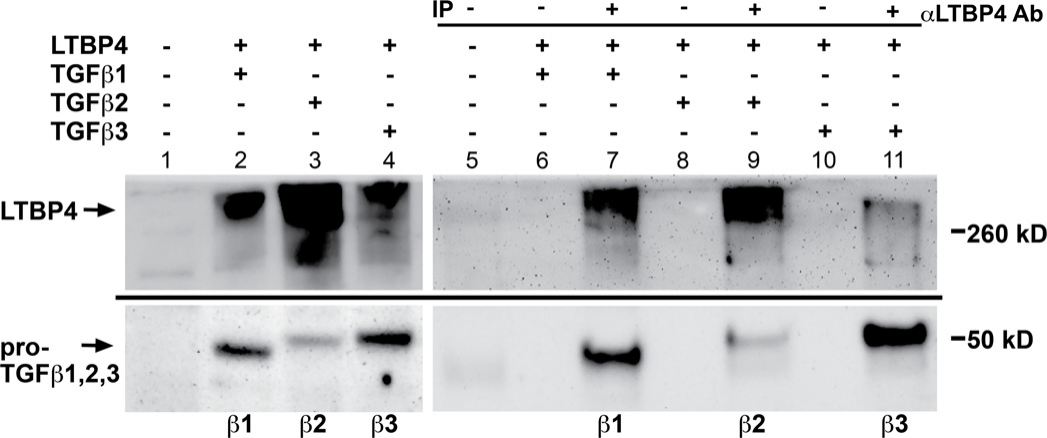
LTBP4 binds TGFβ 1, 2, and 3 isoforms. HEK293T cells were co-transfected with LTBP4 and either TGFβ1, TGFβ2, or TGFβ3. Cell lysates were immunoprecipitated with an antibody to LTBP4 and immunoblotted with an antibody to the FLAG tag on TGFβ1, TGFβ2, and TGFβ3. The immunoblot shows that all three of the TGFβ isoforms associate with LTBP4.

TGFβ’s isoforms TGFβ1, 2 and 3, are expressed in distinct spatiotemporal patterns [32, 33]. Because of the close sequence relationship among TGFβs 1, 2 and 3, we tested each for its ability to interact with LTBP4. HEK293T cells were co-transfected with LTBP4 and plasmids expressing TGFβ1, TGFβ2 or TGFβ3, and immunoprecipitations were carried out using an antibody against LTBP4 followed by immunoblotting for the FLAG-epitope tag on TGFβ1, 2 or 3 (Fig 5C). All three of the TGFβ isoforms demonstrated a clear interaction with LTBP4. TGFβ2 expression was lower than that of TGFβ1 and TGFβ3, which accounts for the weaker binding result of this isoform (lanes 3,9).

## Discussion

LTBP4 is a broadly expressed protein but one that shows higher level of basal expression in cardiac, skeletal and smooth muscle [1, 2]. A mouse model deficient in the short isoform of *Ltbp4* displays early lethality from pulmonary fibrosis, cardiomyopathy and colon cancer, indicating the importance for regulating TGFβ in the heart and during development [30]. *LTBP4* nsSNPs have been linked to a series of diseases including DMD. The two most common *LTBP4* alleles found in the human population differ in four amino acids along the length of the LTBP4 protein. The protective *LTBP4* haplotype correlates with milder disease in DMD, better exercise performance in chronic lung disease, smaller abdominal aneurysms and less invasive colorectal cancer [16, 18–20]. Although deletion of *Ltbp4* in the mouse suggests that LTBP4’s activity is important in the heart, *LTBP4* SNPs had not previously been associated with cardiomyopathy in humans. Although replication studies are needed, a recent study did identify an association between *LTBP4* SNPs and DCM in the setting of DMD. Specifically it was found that steroid-treated DMD patients carrying the rs10880 allele encoding a threonine have a significantly increased incidence of DCM compared to patients homozygous for the protective allele, which encodes a methionine [34]. These findings, coupled with the studies in animal models, suggest a role for LTBP4 in the heart.

Each of these studies examining association between *LTBP4* SNPs and disease phenotypes was carried out in primarily European American populations, including non-Hispanic European Americans from the United States, Sweden, the United Kingdom, New Zealand, and Australia. One report associating *LTBP4* SNPs and ambulation loss in DMD, finding that *LTBP4* genotype was directly associated with age at loss of ambulation [15]. However, this association was found only when the population was stratified to include exclusively non-Hispanic European Americans [15]. In this study, the number of non-European American patients was too small to make any conclusions about the effect of *LTBP4* on age at loss of ambulation in other ethnicities. Despite using a larger non-European American population, we did not find evidence for an association between *LTBP4* SNPs and DCM in African Americans. However, the *LTBP4* locus differs between European Americans and African Americans in its degree of linkage disequilibrium. Specifically, there are fewer crossover events among European Americans and a greater percentage of the European American alleles are VTTT or IAAM versus the deviating genotypes like ITTT, IAAT, and others. These studies, together with the varying linkage disequilibrium across ethnicities, suggest that *LTBP4* genotype effects are more pronounced with preservations of the VTTT/IAAM diplotypes. While it is possibly that the risk alleles are in linkage disequilibrium with other, as yet unidentified genetic variants that explain the phenotypic results, extensive sequencing and examination of the LTBP4 allele has not identified additional nsSNPs that account for this result. African Americans and European Americans differ in their allele frequencies at many sites across the genome, and it is plausible that another missense SNP could affect TGFβ binding or signaling and confound the effect of the VTTT allele in African Americans.

Cells with the *LTBP4* “IAAM” allele display reduced TGFβ signaling compared to cells carrying the “VTTT” allele, despite similar levels of LTBP4 protein [16]. The mechanism underlying this decreased TGFβ signaling is explained by the protective LTBP4 IAAM sequence binding TGFβ more tightly than cells expressing the risk-associated VTTT LTBP4. Increased sequestration of latent TGFβ by matrix-bound LTBP4 results in less release of latent TGFβ and therefore less TGFβ available to bind TGFβ receptors. Reduced TGFβ, whether produced genetically or pharmacologically, has been shown to reduce fibrosis in muscular dystrophy [35–37]. This effect can also be reproduced through a dominant negative TGFβ receptor or through reduction of the canonical TGFβ signaling pathways [38–40]. Noncanonical TGFβ signaling follows this same pattern, with inhibition of noncanonical TGFβ signaling also resulting in an improved muscular dystrophy phenotype [41].

Little is known about the distinct biological roles of the three TGFβ isoforms and their role in disease, although TGFβ1 is the most studied. In DCM, TGFβ1 is upregulated in the plasma and myocardium, however many studies have not examined the expression of TGFβ2 and TGFβ3 [25, 26]. In an unbiased microarray study, TGFβ2 was found to be significantly increased in DCM samples compared to controls [42]. The differential roles of TGFβ isoforms have been studied in several other forms of heart disease and cardiac remodeling. Regulatory variants in *TGFB3* have been associated with arrhythmogenic right ventricular cardiomyopathy [43]. In a rat model of left ventricular hypertrophy all three TGFβ isoforms were induced upon cardiac remodeling, however *TGFB2* expression level increased earlier and was more robust than the other two isoforms [44]. Similarly, in a rat model of myocardial infarction, all three TGFβ isoforms increase in the left ventricle after infarct, however *TGFB2* had the largest fold increase, suggesting that TGFβ2 plays an important role in myocardial remodeling [45]. Notably, this current study examined non-ischemic DCM. TGFβ1, TGFβ2 and, TGFβ3 are co-regulated during wound healing [46]. The varying levels of TGFβ isoform expression, both of which interact with LTBP4, are likely critical during injury and repair.

The association of *LTBP4* SNPs with exercise performance in chronic lung disease could reflect LTBP4 involvement in either smooth and/or striated muscle, as well as in the lung parenchyma itself. The association of *LTBP4* loss of function alleles with early onset pulmonary defects likely supports regulation of TGFβ family members in multiple cell types, especially those critical during development, such as BMPs [47]. The postnatal role of LTBPs, including LTBP4, may be quite distinct and the molecular interactions in differentiated cell types may account for the effect in chronic lung disease, abdominal aneurysms and in colorectal cancer [18–20]. In all of these disorders, it is the *LTBP4* allele associated with hyper-TGFβ signaling and reduced latent TGFβ sequestration that is the risk or deleterious allele. These effects may be explained by the interaction of LTBPs with multiple TGFβ family members.

## Materials and Methods

### Patient populations and controls

Written informed consent was obtained from all human subjects or their parents in the case of minors under the approval of the University of Chicago’s Institutional Review Board. Peripheral blood was collected from unrelated subjects with nonischemic DCM at the University of Chicago Medical Center. Criteria for inclusion included: 1) ejection fraction <45% or 2) left ventricular (LV) end diastolic diameter >117% of the predicted value, and 3) absence of significant epicardial coronary artery disease, and/or hypertensive, systemic, pericardial, or congenital disease to account for DCM [48, 49]. The cohort of 201 subjects included 61 European American and 140 African American DCM subjects, as self-identified. Genomic DNA samples were extracted using the Gentra Puregene Blood Kit (Qiagen #158422). European American and African American control panel DNA was obtained from Coriell (catalog #s HD100CAU and HD100AA-2, respectively). African American control DNA was also obtained through the University of Chicago TRIDOM (Translational Research Initiative in the Department of Medicine) biobank program; samples were selected based on self-identified ethnicity and those with heart disease were excluded. Control DNA from African Americans born at the University of Chicago was extracted from human umbilical cord blood. Echocardiographic data was gathered from a single reading laboratory over 15 years (1999–2014) from 169 subjects. The age at which the echocardiogram was performed was recorded. Ninety-three studies did not list a precise LVEF, classifying LV function as mildly, mildly-moderately, moderately, moderately-severely, or severely reduced. For these studies, the LVEF was translated as 45%, 40%, 35%, 30% and 25%, respectively, based on the reading practices of the laboratory. Six individuals had echocardiography post heart transplant, and therefore were classified as severely reduced LVEF. One individual was estimated as severely reduced LVEF from autopsy records.

### Genotyping

Genomic DNA was amplified by polymerase chain reaction (PCR) for direct sequencing of fragments encompassing SNPs rs2303729, rs1131620, rs1051303, and rs10880 (primers are listed in S4 Table). PCR fragments were purified using ExoSAP-IT reagent (Affymetrix). PCR products were then sequenced with traditional Sanger sequencing and SNP calls were made by analysis of individual chromatograms. DCM patients were separated based on self-identified ethnicity (European American or African American) and allele frequencies were compared with those of ethnicity-matched controls. A chi-square test was used for each SNP to determine whether there was a significant difference in allele frequency between DCM patients and controls. GraphPad Prism was used for statistical analysis.

### Linkage disequilibrium analyses

Linkage disequilibrium maps and haplotype frequencies were constructed in Haploview using the 1000 Genomes EUR super population (n = 503) to represent European Americans and ASW population (n = 61) to represent African Americans [27, 50]. EUR includes CEU (Utah residents with Northern and Western European ancestry, TSI (Toscani in Italy), FIN (Finnish in Finland), GBR (British in England and Scotland), and IBS (Iberian population in Spain). ASW is composed of Americans of African ancestry in the Southwestern United States. The 1000 Genomes VCF to Ped converter was used to obtain data files and locus information files for input into Haploview. SNPs with a population frequency over five percent were included in the map. Linkage disequilibrium blocks were designated using the confidence interval method [51]. The color scheme of the linkage disequilibrium map was generated using the standard D’/LOD option in the Haploview software.

### Expression constructs

Full length human *LTBP4* (NM_001042544.1) and *TGFβ1* (NM_000660.3) cDNA clones were purchased from Origene (catalog numbers SC311430 and SC119746, respectively). *TGFβ2* (NM_003238.3) and *TGFβ3* (NM_003239.2) were amplified by polymerase chain reaction using cDNA from human skin fibroblasts (GM03348, Coriell Cell Repositories) as a template. The FLAG epitope tag (DYKDDDDK) was engineered on the 3’ end of *TGFβ1*, *TGFβ2*, and *TGFβ3* and a Kozak consensus sequence (CACC) was added immediately upstream of the initiation codon. The following primers were used for PCR of TGFβ2 and TGFβ3: TGFβ2 Forward: 5’ CACCATGCACTACTGTGTGCTGAGCGCT 3’; TGFβ2 Reverse: 5’ TCACTTGTCATCGTCATCCTTGTAATCGCTGCATTTGCAAGACTT 3’; TGFβ3 Forward: 5’ CACCATGAAGATGCACTTGCAAAGGGCT 3’; TGFβ3 Reverse: 5’ TCACTTGTCATCGTCATCCTTGTAATCGCTACATTTACAAGACTT 3’. The Xpress epitope (DLYDDDDK) was added onto the 5’/N-terminus of *LTBP4*.

### Cell culture

HEK293-T cells were obtained from ATCC (catalog number CRL-11268). Cells were grown in Dulbecco's Modified Eagle Medium (DMEM) supplemented with 10% fetal bovine serum (Invitrogen, lot #1420768) and 1% penicillin/streptomycin (Invitrogen) in 5% CO_2_.

### Transfections

HEK293-T cells were plated at 1.5x10^6^ cells per well in 6-well plates the day prior to transfection. Media was changed to Opti-MEM Reduced Serum Media one hour prior to transfection. Transfections were performed using FuGENE HD transfection reagent (Promega) at a DNA:FuGENE ratio of 1:3. Opti-MEM was replaced with serum-rich media 24 hours post-transfection and cells were harvested 3 days post-transfection.

### Co-immunoprecipitation

Cultured HEK293T cells were rinsed once with ice-cold phosphate buffered saline and lysed with 150μl lysis buffer (150 mM NaCl, 50 mM Tris HCl (pH 8), 25 mM β-glycerophosphate, 10 mM sodium pyrophosphate, 1x cOmplete Protease Inhibitor Tablet (Roche), 1 mM phenyl-methylsulfonyl fluoride, 2 mM EDTA, and 0.1% Triton X-100), [52]. Cells were scraped from the plate using a chilled cell-lifter and transferred to Eppendorf tubes. Pre-cleared lysates were incubated with 3μg antibody for 3 hours, followed by incubation with 45μl Protein G/A agarose bead slurry (Millipore) for 2 hours. Following this, samples were washed and eluted using 2X Laemmli sample buffer containing β-mercaptoethanol. Pocono Rabbit Farm and Laboratory generated polyclonal anti-LTBP4 and anti-Xpress antibodies using the LTBP4 peptide sequence EPRPEPRPDPRPGPELPLP and anti-Xpress epitope sequence DLYDDDDK.

### Immunoblotting

Prior to SDS-PAGE, 5X Laemmli buffer containing β-mercaptoethanol was added to cell lysates and samples were boiled for five minutes. Fifty μg of protein input (cell lysate pre-IP) and the entire co-IP lysate were separated on a 4–20% polyacrylamide gel (Pierce) and transferred to PVDF Immobilon-P membrane (Millipore). Blocking and antibody incubations were done using StartingBlock T20 (TBS) blocking buffer (Pierce). The membrane was immunoblotted with primary antibody at 1:4000 (anti-FLAG, anti-LTBP4) or 1:500 (anti-Xpress). Mouse monoclonal anti-FLAG was commercially available (Sigma #F1804). Rabbit polyclonal anti-LTBP4 and rabbit polyclonal anti-Xpress antibodies were generated by Pocono Rabbit Farm and Laboratory (details above). Goat anti-mouse and goat anti-rabbit secondary antibodies conjugated to horseradish peroxidase (Jackson Immunoresearch #115-035-003 and #111-035-003) were used at a dilution of 1:8000. Clarity ECL substrate (Bio-Rad) was applied to membranes and membranes were visualized using a Biospectrum Imager (UVP). ImageJ gel analysis tools were used for densitometry analysis. To quantify the amount of immunoprecipitation, immunoprecipitations were performed in triplicate and a student’s t-test was used to test whether the amount of TGFβ immunoprecipitated was significantly different depending on LTBP4 genotype. Prism software (GraphPad) was used for statistical analyses. Uncropped gels are shown in S1 Fig.

## Supporting Information

S1 Fig Uncropped gels for Figs 3, 4 and 5. (PDF)

S1 Table *LTBP4* coordinately expressed genes (top 25). (PDF)

S2 Table KEGG pathways of *LTBP4* coordinately expressed genes. (PDF)

S3 Table Tissue expression of *LTBP4* coordinately expressed genes. (PDF)

S4 Table PCR primers used for genotyping. (PDF)

## Abbreviations

DCM: Dilated cardiomyopathy
DMD: Duchenne muscular dystrophy
HEK: Human embryonic kidney
LTBP4: Latent TGFβ binding protein 4
SNP: Single nucleotide polymorphism
TGFβ: Transforming growth factor β
