# Supplementary figures and images for: Genotype-Specific Interaction of Latent TGFβ Binding Protein 4 with TGFβ
Source: PLoS One. 2016 Feb 26;11(2):e0150358. doi: 10.1371/journal.pone.0150358 (PMC4769137; doi:10.1371/journal.pone.0150358)

S1 Fig

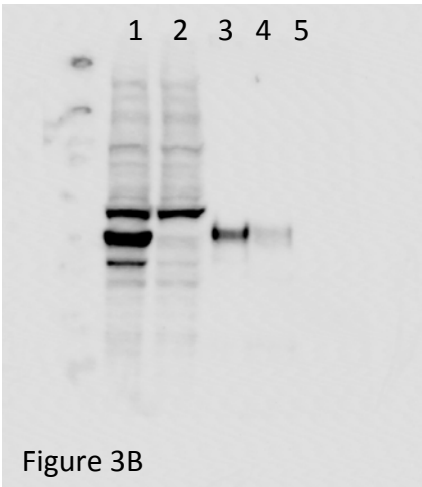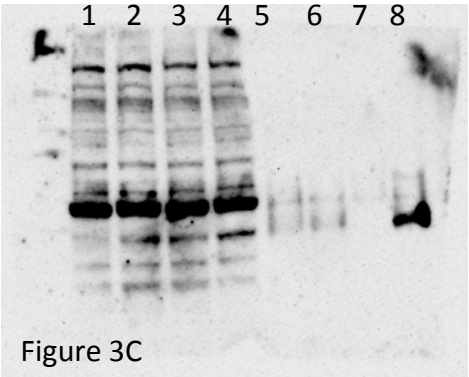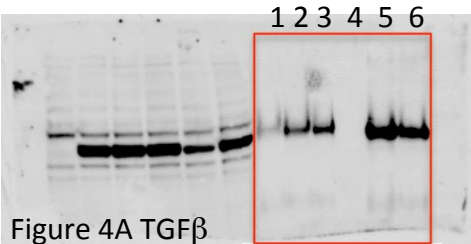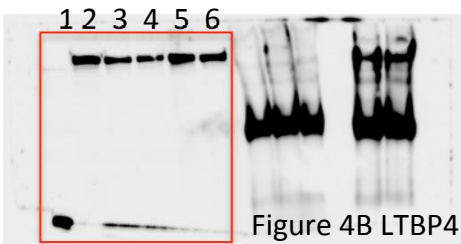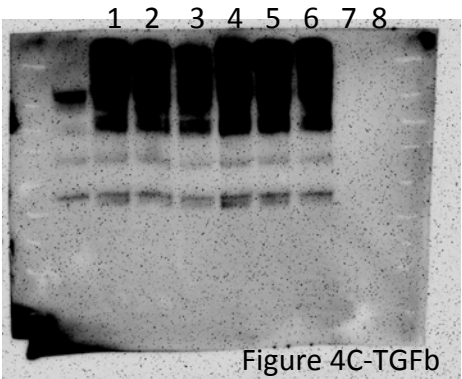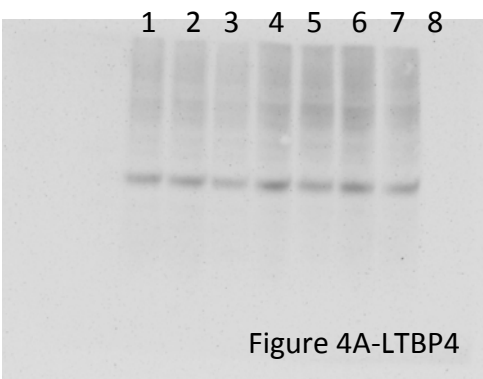

Figure 5 input

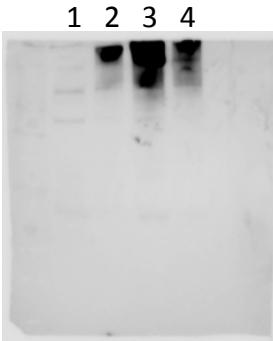

Figure 5 input

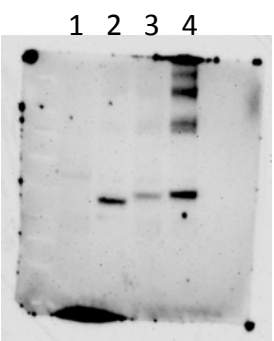

Figure 5 input

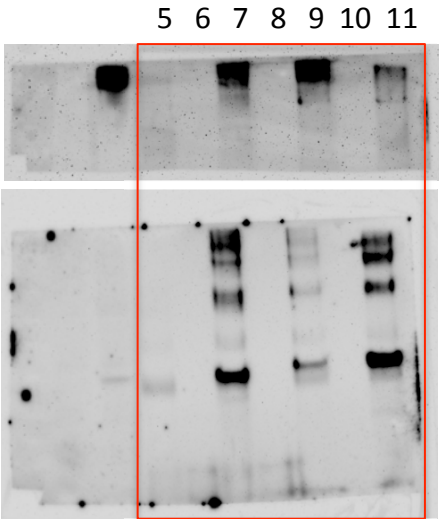

Supplement: S1 Fig — (PDF) [file pone.0150358.s001.pdf]
