# Supplementary material for: Genotype-Specific Interaction of Latent TGFβ Binding Protein 4 with TGFβ
Source: PLoS One. 2016 Feb 26;11(2):e0150358. doi: 10.1371/journal.pone.0150358 (PMC4769137; doi:10.1371/journal.pone.0150358)
Supplement: S1 Table — (PDF) [file pone.0150358.s002.pdf]

S1 Table. *LTBP4* coordinately expressed genes (Top 25).

Gene symbol	Description	# of experiments with LTBP4	%concordant	Pearson Coef	Pearson p-val.
LTBP4	latent transforming growth factor beta binding protein 4	801			
COL6A2	collagen, type VI, alpha 2	483	90.89	0.643	1.39E-94
OGN	osteoglycin	455	87.47	0.609	1.88E-82
COL6A1	collagen, type VI, alpha 1	484	87.81	0.596	2.40E-78
PTGIS	prostaglandin I2 (prostacyclin) synthase	450	88.89	0.572	9.14E-71
TNXB	tenascin XB	395	94.18	0.567	2.91E-69
SSPN	sarcospan	458	87.77	0.566	5.66E-69
COL6A3	collagen, type VI, alpha 3	443	86.46	0.558	1.29E-66
ISLR	immunoglobulin superfamily containing leucine-rich repeat	388	91.75	0.552	4.17E-65
HTRA3	HtrA serine peptidase 3	349	91.12	0.545	3.93E-63
PTRF	polymerase I and transcript release factor	398	91.71	0.545	4.75E-63
DPT	dermatopontin	454	84.14	0.541	5.81E-62
LAMA2	laminin, alpha 2	437	86.27	0.535	2.03E-60
LAMB2	laminin, beta 2 (laminin S)	348	90.23	0.534	2.59E-60
TGFB1I1	transforming growth factor beta 1 induced transcript 1	364	88.74	0.529	5.10E-59
MYH11	myosin, heavy chain 11, smooth muscle	406	86.70	0.525	5.09E-58
TAGLN	transgelin	430	85.58	0.525	7.00E-58
MGP	matrix Gla protein	470	85.32	0.524	9.39E-58
CAV1	caveolin 1, caveolae protein	451	83.81	0.521	6.22E-57
GAS6	growth arrest-specific 6	393	86.77	0.520	1.36E-56
THBS3	thrombospondin 3	245	94.29	0.519	1.47E-56
IGFBP6	insulin-like growth factor binding protein 6	445	85.84	0.514	2.70E-55
ENG	endoglin	337	89.91	0.508	7.17E-54
GSN	gelsolin	446	86.55	0.505	3.82E-53
MFAP4	microfibrillar-associated protein 4	394	87.56	0.500	6.55E-52
KLF2	Kruppel-like factor 2 (lung)	355	83.66	0.499	1.17E-51
