# Supplementary material for: Genotype-Specific Interaction of Latent TGFβ Binding Protein 4 with TGFβ
Source: PLoS One. 2016 Feb 26;11(2):e0150358. doi: 10.1371/journal.pone.0150358 (PMC4769137; doi:10.1371/journal.pone.0150358)
Supplement: S2 Table — (PDF) [file pone.0150358.s003.pdf]

S2 Table. Representative KEGG pathways of *LTBP4* concordant genes.

KEGG Pathway	P-value
ECM-receptor interaction	2.32E-15
Focal adhesion	2.37E-14
Pathways in cancer	0.000225301
Dilated cardiomyopathy	0.000707059
Vascular smooth muscle contraction	0.000829167
Hypertrophic cardiomyopathy (HCM)	0.001681122
Arrhythmogenic right ventricular cardiomyopathy (ARVC)	0.003173175
DNA replication	0.012518785
Small cell lung cancer	0.024163892
TGF-beta signaling pathway	0.026731043
Cell cycle	0.041140391
