# Supplementary material for: Genotype-Specific Interaction of Latent TGFβ Binding Protein 4 with TGFβ
Source: PLoS One. 2016 Feb 26;11(2):e0150358. doi: 10.1371/journal.pone.0150358 (PMC4769137; doi:10.1371/journal.pone.0150358)
Supplement: S3 Table — (PDF) [file pone.0150358.s004.pdf]

S3 Table. Tissue expression of *LTBP4* coordinately expressed genes.

**Tissue**	**P-value**
Heart	2.43E-12
Placenta	3.00E-09
Plasma	3.53E-09
Liver	1.14E-06
Brain	1.22E-06
Aorta	1.41E-06
Lung	6.09E-06
Uterus	2.40E-05
Fibroblast	9.77E-05
Adipocyte	0.001667018
Cartilage	0.001937652
Fetal lung	0.002123478
Synovial membrane	0.002567117
Colon tumor	0.003405925
Platelet	0.003686038
Skeletal muscle	0.003759569
White Matter pool- 5 brain tissues- femoral artery	0.006170416
Embryo	0.011716358
Umbilical vein endothelial cell	0.01208514
Pancreas	0.012565667
Fetal brain cortex	0.017828712
Epithelium	0.021593582
Kidney	0.025073738
Bone marrow	0.02598837
Foreskin	0.027471571
Synovial membrane tissue	0.030804699
Umbilical vein	0.035979968
Cancellous bone	0.041333335
PNS	0.044367471
Muscle	0.048099421
