# Supplementary material for: Genotype-Specific Interaction of Latent TGFβ Binding Protein 4 with TGFβ
Source: PLoS One. 2016 Feb 26;11(2):e0150358. doi: 10.1371/journal.pone.0150358 (PMC4769137; doi:10.1371/journal.pone.0150358)
Supplement: S4 Table — (PDF) [file pone.0150358.s005.pdf]

S4 Table. PCR primers used to genotype dilated cardiomyopathy patients and controls for *LTBP4* SNPs.

Single nucleotide polymorphism	Forward primer	Reverse primer
**rs2303729**	AGGAGAACCTGCCAGGAATGG	GGGGCTAGACAAGGCGGGGA
**rs1051303 and rs1131620**	GAATGGATTCAGGCCCCTTCCTCAGCCTCATT	ATTATGAACCCAACCAGGTTCCAGAGAA
**rs10880**	TGAGAGGTGTGGAGTCTGGTTCTGCCA	CAGTGCACACCCTGTGAACATTTGTTGA
